# Eotaxin-1/CCL11 is involved in cell migration in rheumatoid arthritis

**DOI:** 10.1038/s41598-021-87199-7

**Published:** 2021-04-12

**Authors:** Kuninobu Wakabayashi, Takeo Isozaki, Yumi Tsubokura, Sayaka Fukuse, Tsuyoshi Kasama

**Affiliations:** Division of Rheumatology, Department of Medicine, Showa University School of Medicine, 1-5-8 Hatanodai, Shinagawa-ku, Tokyo, Japan

**Keywords:** Immunology, Rheumatology

## Abstract

Eotaxin-1 (CCL11) induces the migration of different leukocyte types by interacting with CCR3. In rheumatoid arthritis (RA), fibroblast-like synoviocytes (FLS) are pathogenic effectors and a major CCR3-expressing cell. The aim of this study was to investigate the expression and function of CCL11 in RA FLS. The expression of CCL11 and CCR3 was evaluated by ELISA, immunofluorescence and quantitative PCR analysis. The CCL11 levels in serum and synovial fluids (SFs) from RA patients were significantly higher than those in serum from healthy controls and SFs from osteoarthritis patients. CCL11 and CCR3 were expressed in the RA synovial tissue lining layers. The secretion of CCL11 in RA FLS-conditioned medium and the mRNA expression of CCL11 and CCR3 were induced by TNF-α. Furthermore, CCL11 induced the mRNA expression of CCL11 and CCR3. Application of a CCR3 antagonist reduced TNF-α-induced CCL11 secretion from RA FLS. CCL11 induced the migration of RA FLS and monocytes. RA FLS migration was decreased by treatment with CCL11 siRNA. The migration of monocytes to medium conditioned with CCL11 siRNA-transfected and TNF-α-stimulated RA FLS was reduced. These data indicate that the self-amplification of CCL11 via CCR3 may play an important role in cell migration in RA.

## Introduction

Rheumatoid arthritis (RA) is the most common chronic autoimmune disease characterized by inflammatory synovitis and hyperproliferation of synovial cells leading to progressive destruction of cartilage and bone in multiple joints^1^. The fibroblast-like synoviocytes (FLS) present in the intimal lining of the synovium promote inflammation and joint destruction in RA^2,3^. RA FLS participate in bone erosion formation by directly invading articular cartilage and secreting matrix metalloproteinases that break down the extracellular matrix and cartilage. RA FLS also contribute to the inflammation through the secretion of cytokines and chemokines which induce and maintain the inflammatory cells, including infiltrating lymphocytes and monocytes. It has been suggested that combination of agents selectively targeting RA FLS and current disease-modifying anti-rheumatic drugs is an option for therapy to improve disease control without increasing the risk of infection^3–5^.

Chemokine C–C motif ligand 11 (CCL11), also known as eotaxin-1, is a member of the CC chemokine family^6^. Human CCL11 is produced by connective tissue cells and leukocytic cells. The expression of CCL11 on epithelial cells and fibroblasts has been investigated, and it has been shown that CCL11 is induced by pro-inflammatory cytokines such as tumor necrosis factor-α (TNF-α)^7–9^. CCL11 binds to the C–C chemokine receptors CCR3 with the highest affinity, and also bind to CCR2 and CCR5^10,11^. Through interaction with CCR3, CCL11 induces the migration of several types of leukocytes including eosinophils, basophils, macrophages and dendritic cells^12–16^. CCL11 also induces chemotaxis of endothelial cells and promotes angiogenesis^17,18^.

It has been reported that the concentration of CCL11 in patients with RA before disease onset was significantly higher than that in healthy controls, and it increased further after the onset of RA^19^. RA FLS have been shown to be one of the major populations of CCR3-expressing cells in the synovial tissue (ST). CCR3 mRNA expression in RA FLS is induced by CCL11 stimulation, and IL-1β induces CCL11 release from RA FLS^20^. A recent study revealed that the receptor activator of nuclear factor kappa-B ligand stimulated CCR3 expression in osteoclasts and that the addition of CCL11 caused an increased migration of preosteoclasts and an increase in osteoclastic bone resorption^21^. However, the role of CCL11 in RA synovium is unclear. The aim of this study was to investigate the expression and function of CCL11 and its relationship to other CC chemokines in RA FLS.

## Results

### CCL11 is expressed in serum and SFs from RA

**Figure 1:**
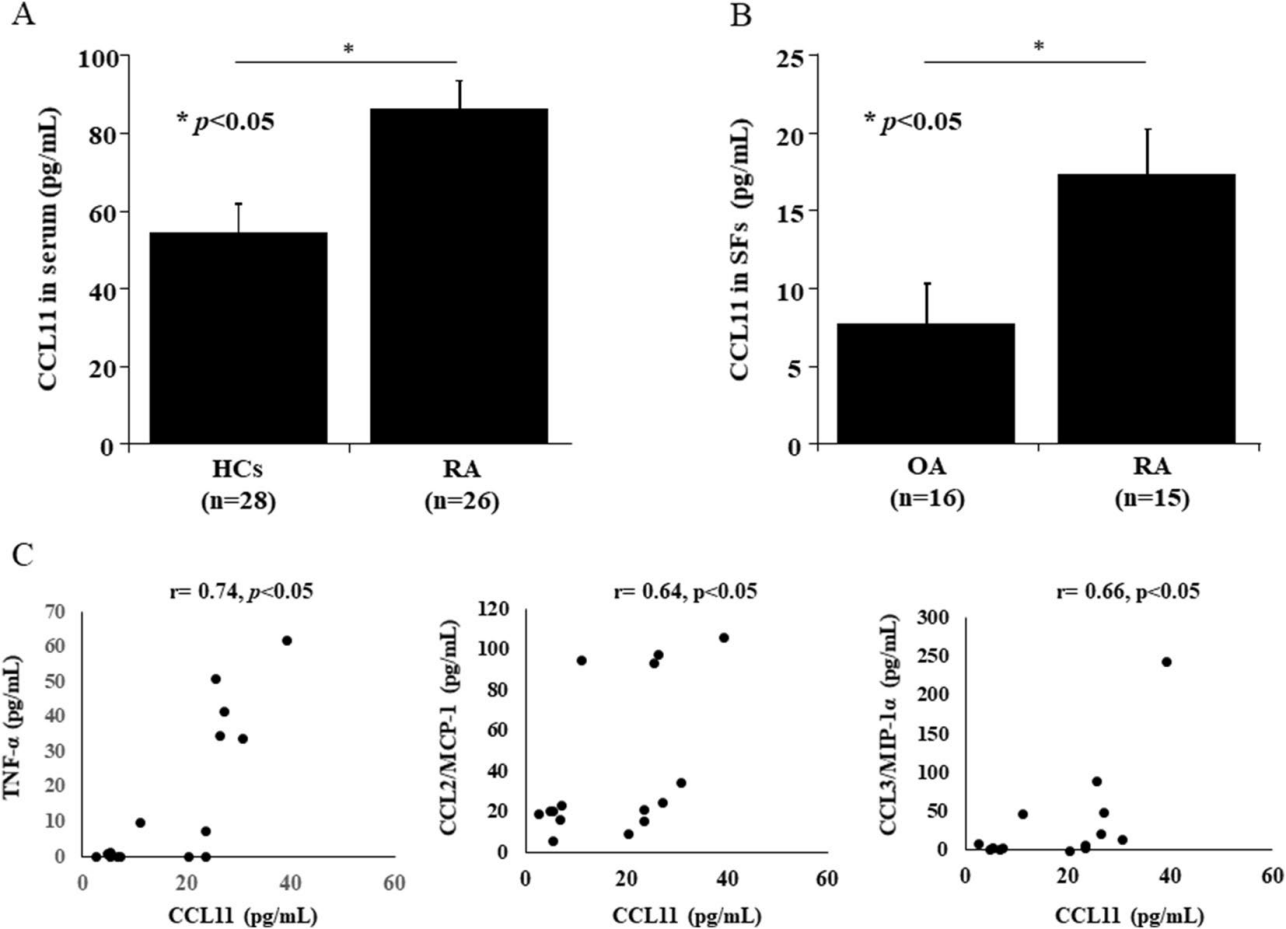
CCL11 is expressed in serum and synovial fluids (SFs) from rheumatoid arthritis (RA) patients. (**A**) The levels of CCL11 in the serum from RA patients (n = 26) were higher than those in the serum from healthy controls (HCs) (n = 28). (**B**) The levels of CCL11 in SFs from patients with RA (n = 15) were higher than those in SFs from patients with osteoarthritis (OA) (n = 16). The data values are expressed as the mean ± SEM. (**C**) The CCL11 levels were positively correlated with the levels of TNF-α, CCL2/MCP-1 and CCL3/MIP-1α in SFs of RA patients. *p < 0.05 was significant.

We investigated the levels of CCL11 in serum and SFs at the onset of RA using ELISA. The levels of CCL11 in the serum from RA patients (n = 26) were higher than those in the serum from healthy controls (HCs) (n = 28) (mean ± SEM: 86.4 ± 7.0 pg/mL and 54.2 ± 7.5 pg/mL, respectively, *p* < 0.05, Fig. 1A). The levels of CCL11 in SFs from patients with RA (n = 15) were higher than those in SFs from patients with OA (n = 16) (mean ± SEM: 17.2 ± 3.0 pg/mL and 7.7 ± 2.6 pg/mL, respectively, *p* < 0.05, Fig. 1B). We also measured the levels of TNF-α and a few major CC-chemokines, CCL2, CCL3, and CCL5, whose expression was increased by TNFα from RA FLS^22,23^. The CCL11 levels were positively correlated with the levels of TNF-α (r = 0.74, *p* < 0.05), CCL2 (r = 0.64, *p* < 0.05) and CCL3 (r = 0.66, *p* < 0.05) (Fig. 1C). The levels of CCL5/RANTES were not correlated with the levels of CCL11 (r = 0.20, *p* = 0.46).

### CCL11 and CCR3 are expressed in RA ST and RA FLS

**Figure 2:**
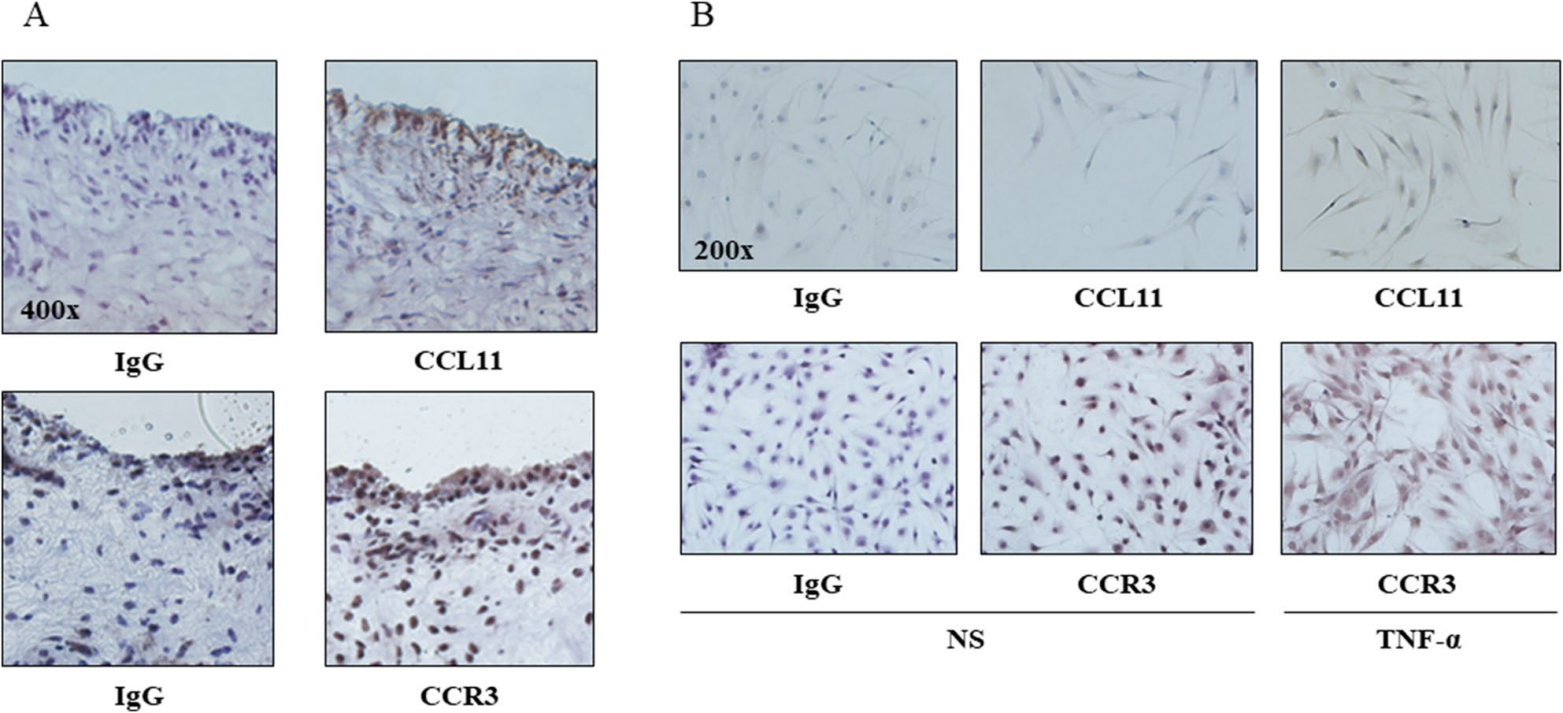
Immunohistochemistry showed that CCL11 and CCR3 were expressed in rheumatoid arthritis (RA) synovial tissues (STs) and fibroblast-like synoviocytes (FLS). (**A**) CCL11 and CCR3 were observed in the RA STs lining layers (magnification × 400). (**B**) CCL11 and CCR3 were expressed in RA FLS and were increased in FLS stimulated with 50 ng/mL TNF-α compared with the unstimulated FLS (magnification × 200). *NS *no stimulation.

**Figure 3:**
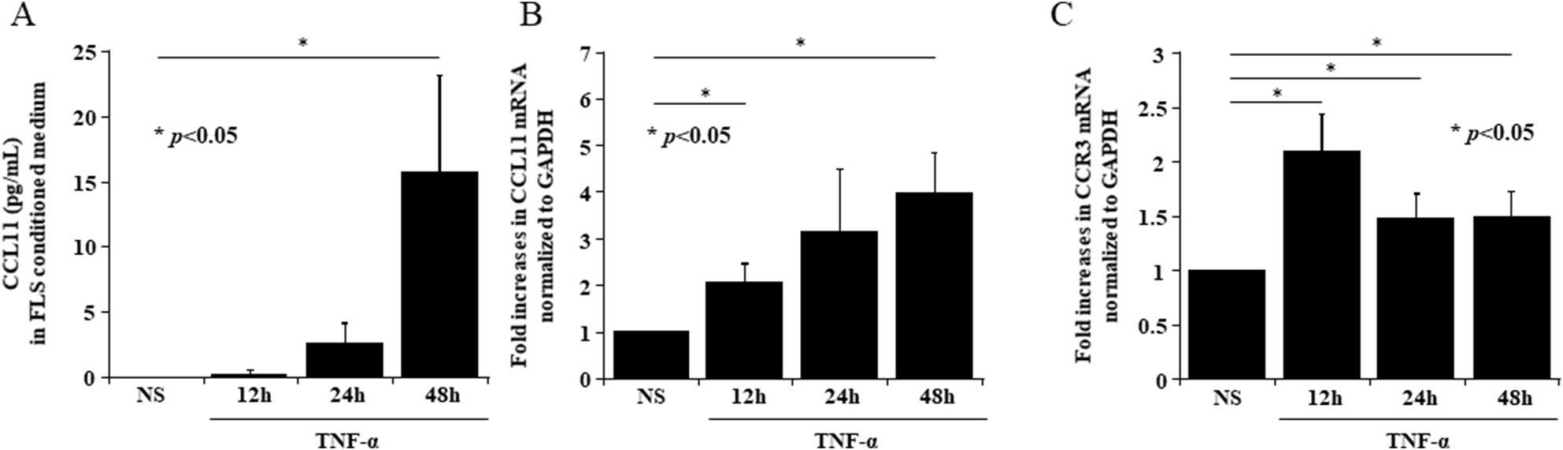
TNF-α stimulation increased the expression of CCL11 and CCR3 in RA FLS. (**A**) The secretion of CCL11 in RA FLS-conditioned medium and (**B**) the expression of CCL11 mRNA were increased by 50 ng/mL TNF-α stimulation in a time-dependent manner at 12, 24 and 48 h (n = 7 patients). (**C**) The peak expression of CCR3 mRNA induced by 50 ng/mL TNF-α stimulation was at 12 h (n = 7 patients). *NS *no stimulation. The data values are expressed as the mean ± SEM. *p < 0.05 was significant.

We measured the expression of CCL11 and CCR3 in RA ST using immunohistochemistry. We found that CCL11 and CCR3 were expressed in RA cells lining STs (Fig. 2A). Next, we assessed CCL11 and CCR3 expression in RA FLS using immunohistochemistry. We demonstrated that CCL11 and CCR3 were expressed in RA FLS and that their expression levels were increased by recombinant TNF-α stimulation (Fig. 2B). We then measured the secretion of CCL11 in RA FLS-conditioned medium using ELISA and the mRNA expression of CCL11 and CCR3 using qPCR. The secretion of CCL11 in RA FLS-conditioned medium (Fig. 3A) and the expression of CCL11 mRNA (Fig. 3B) were increased by TNF-α stimulation in a time-dependent manner at 12, 24 and 48 h (n = 7 patients). The peak expression of CCR3 mRNA was induced by TNF-α stimulation at 12 h (n = 7 patients, Fig. 3C).

### CCL11 stimulation induces CCL11 and CCR3 mRNA

**Figure 4:**
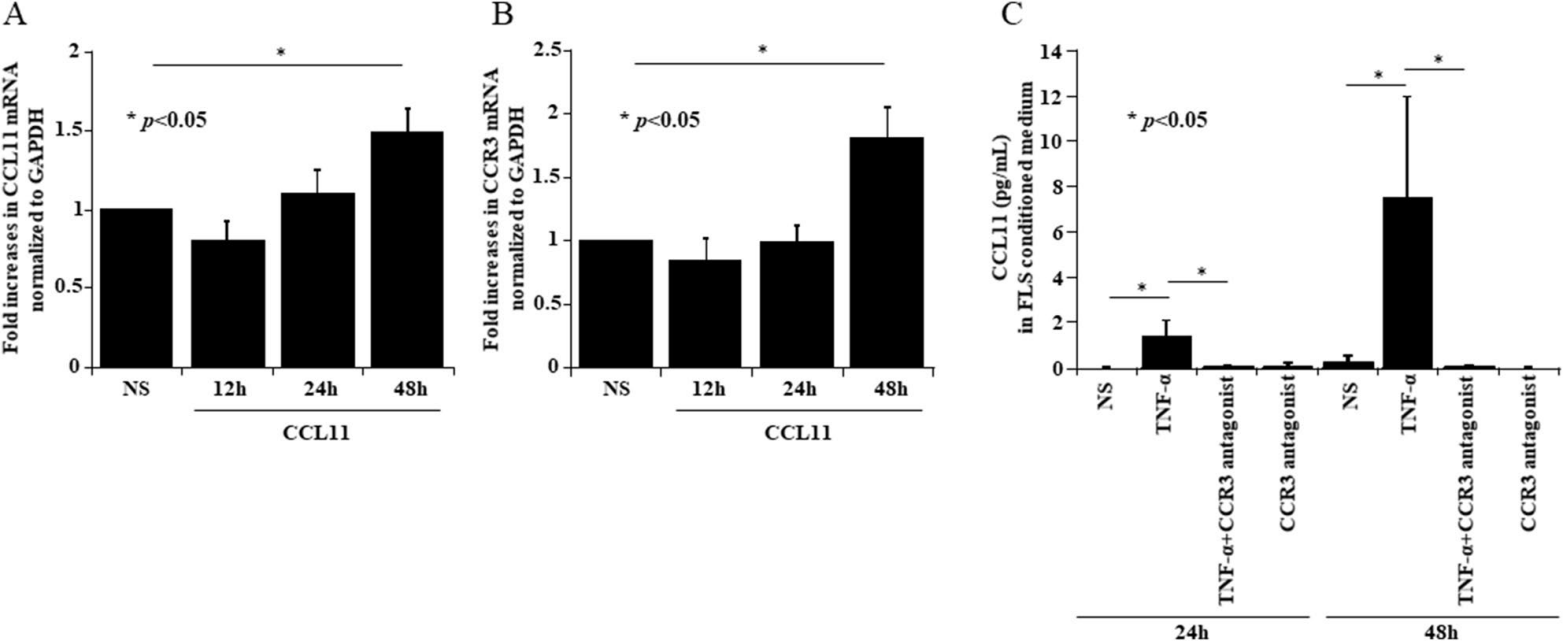
CCL11 stimulation induced CCL11 and CCR3 mRNA. (**A**) RA FLS were stimulated with 200 ng/mL CCL11 for 12, 24 and 48 h. The expression of CCL11 mRNA at 48 h was induced by CCL11 self-stimulation (n = 5 patients). (**B**) The expression of CCR3 mRNA at 48 h was induced by CCL11 stimulation (n = 5 patients). (**C**) An antagonist of CCR3 (SB328437) at 100 µM reduced TNF-α-induced CCL11 secretion from RA FLS (n = 3 patients). *NS *no stimulation. The data are expressed as the mean ± SEM. *p < 0.05 was significant.

Because CCL11 expression was increased by TNF-α stimulation in a time-dependent manner, we hypothesized that CCL11 expression was induced by CCL11 itself and/or other factors. We assessed the mRNA expression of CCL11 and CCR3 in recombinant CCL11-stimulated RA FLS using qPCR. The expression of CCL11 mRNA at 48 h was induced by CCL11 self-stimulation compared with control treatment (no stimulation) (Fig. 4A). The expression of CCR3 mRNA at 48 h was also induced by CCL11 stimulation compared with control treatment (Fig. 4B). We then measured the effect of a CCR3 antagonist (SB328437) on the secretion of CCL11 in TNF-α-stimulated RA FLS-conditioned medium. Treatment with the CCR3 antagonist reduced the TNF-α-induced CCL11 secretion from RA FLS (Fig. 4C).

### CCL11 induced the migration of RA FLS

**Figure 5:**
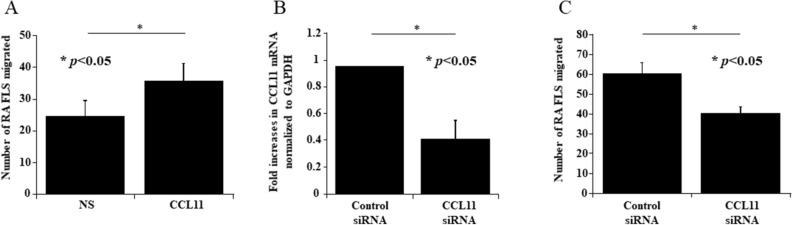
CCL11 induced the migration of RA FLS and THP-1 cells. (**A**) CCL11-stimulated RA FLS were significantly more efficient at migration than unstimulated FLS (n = 3 patients, 3 experiments each). (**B**) CCL11 siRNA treatment decreased the expression of CCL11 mRNA (n = 3 patients). (**C**) CCL11 siRNA treatment decreased the migration of RA FLS (n = 2 patients, 3 experiments each). *NS *no stimulation. The data are expressed as the mean ± SEM. *p < 0.05 was significant.

To determine whether CCL11 induced the migration of RA FLS, we performed transwell migration assays. CCL11-stimulated cells migrated significantly more efficiently than unstimulated cells (mean ± SEM, number of cells per field; 35.8 ± 5.2 and 24.5 ± 4.6, respectively, *p* < 0.05, Fig. 5A). We next explored the possibility that reduced expression of CCL11 could inhibit the migration of RA FLS. We used siRNA directed against CCL11. The knockdown of CCL11 in RA FLS was confirmed by the mRNA expression of CCL11, which was lower than that in control cells (Fig. 5B). We found that CCL11 siRNA-treated RA FLS showed decreased migration toward 5% FBS compared with control siRNA-treated cells (mean ± SEM, number of cells per field; 40.0 ± 3.6 and 60.0 ± 5.6, respectively, *p* < 0.05, Fig. 5C).

### CCL11 induced the migration of THP-1 cells

**Figure 6:**
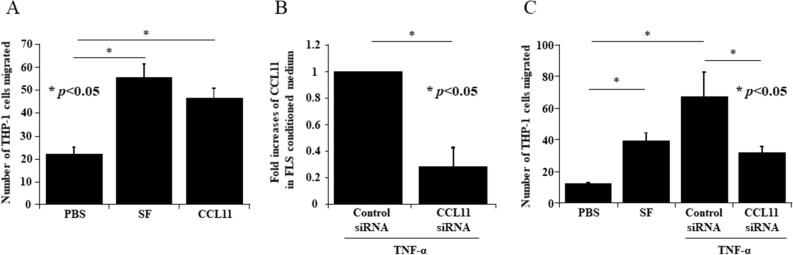
CCL11 induced the migration of THP-1 cells. (**A**) CCL11-stimulated THP-1 cells were significantly more efficient at migration than unstimulated cells (n = 4 experiments). *SF* synovial fluid. (**B**) CCL11 siRNA treatment decreased the secretion of CCL11 in TNF-α-treated RA FLS-conditioned medium (n = 3 patients). (**C**) CCL11 siRNA-transfected and TNF-α-stimulated RA FLS-conditioned medium reduced THP-1 migration compared to control siRNA-transfected and TNF-α-stimulated RA FLS-conditioned medium (n = 4 patients). The data are expressed as the mean ± SEM. *p < 0.05 was significant.

Next, we performed in vitro THP-1 chemotaxis assays to determine whether CCL11 induced monocyte migration. The number of cells migrating toward CCL11 was significantly larger than the number of cells migrating toward the control (mean ± SEM, number of cells per field; 46.5 ± 4.2 and 22.0 ± 2.9, respectively, *p* < 0.05, Fig. 6A). To confirm the effect of siRNA treatment against CCL11 in RA FLS, we evaluated THP-1 cell chemotaxis toward medium conditioned by CCL11 siRNA-transfected and TNF-α stimulated RA FLS. The knockdown of CCL11 in RA FLS-conditioned medium was confirmed by ELISA. The secretion of CCL11 in CCL11 siRNA-transfected and TNF-α-stimulated RA FLS-conditioned medium was lower than that in control siRNA-transfected RA FLS-conditioned medium (n = 3, Fig. 6B). In addition, the number of cells that migrated toward CCL11 siRNA-transfected and TNF-α stimulated RA FLS-conditioned medium was lower than the number that migrated toward control siRNA-transfected and TNF-α stimulated RA FLS-conditioned medium (mean ± SEM, number of cells per field; 31.9 ± 3.6 and 66.9 ± 16.0, respectively, *p* < 0.05, Fig. 6C).

## Discussion

In this study, we demonstrated that the levels of CCL11 in serum from RA patients were higher than those in the serum from HC, and the levels of CCL11 in SFs from patients with RA were higher than those in SFs from patients with OA. Furthermore, the CCL11 levels in SFs were positively correlated with the levels of TNF-α, CCL2, and CCL3. These data suggest that CCL11, like other CC chemokines, is involved in joint inflammation in RA. We measured the concentration of CCL11 in serum and SF from the patients without any treatment at the time of diagnosis. We also collected both SF and serum from 15 patients at same day. The levels of CCL11 in SF were lower than those in serum. However, the levels of CCL11 in SF were positively correlated with the levels of TNF-α and the serum levels of CCL11 were not. Therefore, we think the CCL11 in SF is more useful inflammatory mediator than that in serum. These results were different from previous study reported by Liu et al.^20^. We guess the serum CCL11 level is affected by some factors other than arthritis. We compared the concentration of serum CCL11 from 16 patients at pretreatment and post treatment at 6 months, and we could not find any significant differences. And serum CCL11 levels in about a half of patients were increased. We guess serum levels of CCL11 might be affected by treatment or disease duration. Syversen et al. reported that higher serum levels of CCL11 predicted less radiographic progression in early RA patients^24^. Sohn et al. reported that serum CCL11 level is associated with radiographic spinal damage in patients with ankylosing spondylitis^25^. On the other hand, Kindstedt et al. showed CCL11 is a novel mediator of inflammatory osteoclastic bone resorption^21^. The role of CCL11 at the bone resorption or bone formation in the RA patients is currently unclear. Future studies should be performed to confirm whether CCL11 levels in serum or SF at pretreatment predict radiographic progression. CCL11 and CCR3 were expressed in RA FLS and were increased by TNF-α stimulation. Liu et al. reported that TNF-α reduced CCL11 release^20^. This result is different from ours. Although there are individual differences in CCL11 secretion from RA FLS by TNF-α stimulation, we demonstrated TNF-α increased CCL11 secretion in a time-dependent manner using 7 patients. And we also demonstrated significant increase of CCL11 by TNF-α stimulation at 24 h using the RA FLS from 3 patients which secrete CCL11 well in the experiment using CCR3 antagonist. RA FLS are one of the cellular sources of CCL11 and are also susceptible to CCL11 stimulation in the inflammatory environment of RA. The time course study showed that CCL11 induced the expression of CCR3 and also its own expression. Inhibition of TNF-α-induced CCL11 secretion from RA FLS by a CCR3 antagonist indicates that TNF-α-induced CCL11 expression depends on activation of CCR3 and ligands of CCR3 including CCL11. It has been reported previously that CCL11 upregulates the expression of CCR3 mRNA, and CCL11-induced CCR3 expression is reduced by a CCR3 antagonist in RA FLS^20^. Therefore, CCR3 plays an important role in the secretion of CCL11 from RA FLS. Recombinant CCL11 induced the migration of RA FLS, and the migration of RA FLS treated with siRNA against CCL11 decreased. These results suggest CCL11 from RA FLS might induce the migration of RA FLS. CCL11 induced the migration of monocytes, and the migration of monocytes to CCL11 siRNA-transfected and TNF-α-stimulated RA FLS-conditioned medium was reduced. Therefore, CCL11 contributes to cell recruitment to RA joint inflammation. RA FLS are key mediators of inflammation and joint destruction in RA. These cells aggressively invade the extracellular matrix, producing proteases and inflammatory cytokines and chemokines. Upon TNF-α stimulation, CCL11 secreted from RA FLS should bind to CCR3, which is also overexpressed, and induce the upregulation of CCL11 and CCR3 expression on RA FLS. This self-amplification mechanism of CCL11 via CCR3 may contribute to aggressive behavior of RA FLS.

In conclusion, the increased levels of CCL11 in the serum and SF, enhanced expression of CCL11 and CCR3 on RA FLS induced by TNF-α, and induction of monocyte and RA FLS migration by CCL11 indicate that CCL11 plays an important role in the pathogenesis of RA. The suppression of CCL11 and CCR3 expression could be one of the therapeutic effects of anti-TNF agents on RA FLS-induced inflammation in RA.

## Methods

### Patients

RA ST samples were obtained from patients undergoing arthroplasty. The patients with RA were diagnosed using the American College of Rheumatology 1987 revised criteria for RA. Serum was collected from 26 patients with RA before the initial treatment and from 28 healthy controls. The healthy controls were Japanese blood donors matched for gender and age. SFs were collected from 15 patients with RA before the initial treatment and 16 patients with osteoarthritis (OA). All specimens were obtained with informed consent and collected following approval from the Showa University Institutional Review Board. All methods were carried out in accordance with relevant guidelines and regulations.

### Cell culture

Fresh ST samples were minced and digested in tissue enzyme digestion solution as described previously^26^. FLS were cultured in RPMI-1640 medium supplemented with 10% fetal bovine serum (FBS) (Omega Scientific), 100 units/mL penicillin, and 100 µg/mL streptomycin at 37 °C in a humidified 5% CO_2_ atmosphere. The cells were seeded in 6-well plates (BD Biosciences, Bedford, MA, USA) at a density of 2 × 10^5^ cells per well and were maintained in complete medium. For all experiments in this study, FLS were used between passages 4 and 9, and cells were synchronized in 0.1% bovine serum albumin (BSA) (serum starvation medium) for 24 h prior to analysis or functional assays.

THP-1 cells (a human acute monocyte leukemia cell line) were purchased from the American Type Culture Collection (Manassas, VA, USA). THP-1 cells were cultured in complete RPMI 1640 medium as described previously.

### Enzyme-linked immunosorbent assay (ELISA)

ELISAs were performed as described previously^27^. The levels of CCL11 in the cell supernatants of RA FLS-conditioned medium stimulated with 50 ng/mL TNF-α (R&D Systems, Minneapolis, MN, USA) with or without 100 µM of the specific CCR3 antagonist SB328437 (Abcam, Cambridge, MA, USA) were measured using an ELISA kit (R&D Systems) following the manufacturer’s protocol. The plates were developed using tetramethylbenzidine as a substrate (TMB, Sigma-Aldrich, ST. Louis, MO, USA), and the absorbance was recorded using a microplate reader. The levels of CCL11, MCP-1/CCL2, MIP-1α/CCL3, RANTES/CCL5 and TNF-α in serum and SFs were also measured using ELISA kits (R&D Systems).

### Quantitative real-time reverse transcription polymerase chain reaction (PCR)

Following cell synchronization for 24 h, cells were left unstimulated or were stimulated for 12, 24 or 48 h with 50 ng/mL TNF-α. Total RNA was extracted using RNeasy Mini RNA isolation kits (Qiagen, Valencia, Spain) in accordance with the manufacturer’s protocol. Complementary DNA (cDNA) was synthesized using a High-Capacity cDNA Reverse transcription Kit (Applied Biosystems, Waltham, MA, USA) per the manufacturer’s protocol. CCL11, CCR3, and glyceraldehyde 3-phosphate dehydrogenase (GAPDH) primers were purchased from Qiagen. Quantitative PCR was performed using SYBR Green quantitative PCR master mix (Qiagen) on a C1000 thermal cycler (Bio Rad, Hercules, CA, USA) as described previously^5^. The efficiency of the primer assays was guaranteed by the manufacturer to be > 90%. Each reaction was measured in triplicate, and the data were normalized to the expression levels of the housekeeping gene GAPDH. The ratio of each mRNA relative to the GAPDH mRNA was calculated using the ΔΔ threshold cycle method.

### Immunohistochemical analysis

Frozen sections of RA ST and RA FLS isolated from ST were stained for CC11 or CCR3 by the immunoperoxidase method as described previously^28^. The slides were fixed in cold acetone for 20 min and washed with phosphate-buffered saline (PBS). Following incubation with 3% H_2_O_2_ for 10 min to block endogenous peroxidase, the ST samples were blocked with 20% FBS and 5% goat serum in PBS for 1 h at 37 °C. We also stained RA FLS that were left unstimulated or were stimulated for 48 h with 50 ng/mL TNF-α. Rabbit anti-human CCL11 antibody, rabbit anti-human CCR3 antibody (Abcam, Cambridge), and rabbit IgG (Santa Cruz Biotechnology, Santa Cruz, CA, USA) were used as primary antibodies. The slides were incubated overnight at 4 °C. The ST samples were washed with PBS, and biotinylated goat anti-rabbit IgG (Vector Laboratories, Burlingame, CA, USA) was added as a secondary antibody. The slides were incubated for 1 h at 37 °C. After washing, antibody binding was detected with the Vectastain Avidin–Biotin Complexstandard kit (Vector Laboratories) and 3,3′-diaminobenzidine (DAB; Vector Laboratories) as the chromogen. Finally, the slides were counterstained with Gill’s hematoxylin and washed with a series of 70%, 95%, and 100% ethanol and 100% isopropyl alcohol. The images of STs were captured at 400 × magnification, and the images of RA FLS were captured at 200 × magnification.

### Transfection of RA FLS with CCL11 small interfering RNA (siRNA)

RA FLS were seeded in 6-well plates at a density of 2 × 10^5^ cells per well. The siRNA (50 nM) against CCL11 or control siRNA (Santa Cruz Biotechnology) was mixed with TransIT-TKO transfection reagent (Mirus, Madison, WI, USA) according to the manufacturer’s instructions and overlaid on the cells. The cells were incubated with siRNA/TransIT-TKO for 24 h at 37 °C. Knockdown of CCL11 secretion in TNF-α-stimulated FLS-conditioned medium was confirmed using ELISA.

### Transwell migration assays for RA FLS

The transwell migration assays were performed in Transwell systems. RA FLS (5 × 10^5^) were resuspended in assay media (RPMI with 0.5% BSA) with 200 ng/mL CCL11 and allowed to migrate through uncoated transwell chambers in response to 5% FBS for 4 h. Equal numbers of live RA FLS (5 × 10^5^) that were pretreated with CCL11 siRNA or control siRNA for 24 h were resuspended in only assay media and allowed to migrate through uncoated transwell chambers in response to 5% FBS for 4 h. After 4 h, cells were stained with DAPI (Life Technologies, Carlsbad, CA, USA) for 20 min at room temperature. The fluorescence of the migrating cells on each membrane was visualized using an Olympus microscope IX71. Images were acquired from four nonoverlapping fields per membrane, and invading cells in each field were counted visually. Each experiment included four membranes per sample.

### Chemotaxis assays for THP-1

Chemotaxis assays were performed as described previously^28^. For the THP-1 chemotaxis assays, a 48-well Boyden chamber with a 5 μm polycarbonate membrane was used. The lower wells were filled with the stimulus solution. SF [1:50 dilution (in 0.5% BSA/RPMI) was used as the positive control, and 0.5% BSA/RPMI was used as the negative control. THP-1 cells in 0.5% BSA/RPMI at 1.2 × 10^6^/mL were added to the upper wells and incubated at 37 °C for 90 min in an incubator. After incubation, the membrane was stained with Diff-Quick. The migrated cells were counted by a blinded observer. Three high-power (400 ×) fields were counted in each well, and the results were expressed as the number of cells per high-power field.

### Statistical analysis

The data were analyzed using Student’s *t* test for normally distributed variables and the Mann–Whitney *U* test for non-normally distributed variables. The data are reported as the mean ± standard error of the mean (SEM). A *p* value less than 0.05 was considered statistically significant.

## Data Availability

The data that support the findings of this study are available from the corresponding author upon request.
